# Association of secreted frizzled-related protein 4 (SFRP4) with type 2 diabetes in patients with stable coronary artery disease

**DOI:** 10.1186/s12933-014-0155-2

**Published:** 2014-11-19

**Authors:** Michael M Hoffmann, Christian Werner, Michael Böhm, Ulrich Laufs, Karl Winkler

**Affiliations:** Institute of Clinical Chemistry and Laboratory Medicine, University Medical Center Freiburg, Freiburg, Germany; Klinik für Innere Medizin III, Universitätsklinikum des Saarlandes, Homburg, Germany

**Keywords:** SFRP4, Type 2 diabetes mellitus, Coronary artery disease

## Abstract

**Background:**

Secreted frizzled-related proteins (SFRP) are regulators of Wnt-signalling. SFRP4 has been shown to regulate insulin exocytosis and is overexpressed in type 2 diabetes mellitus.

Here we characterized the relation of SFRP4 to glucose and triglyceride metabolism and outcomes in patients with stable coronary artery disease on statin treatment in the prospective Homburg Cream & Sugar Study (NCT00628524).

**Methods:**

Fasting SFRP4 concentrations were measured by ELISA in 504 consecutive patients with stable CAD confirmed by angiography.

**Results:**

The median age was 68 years and 83% of patients were male. Oral glucose tolerance tests were performed in all patients without known diabetes for metabolic characterization. 24.4% of patients showed normal glucose tolerance, 29.4% impaired glucose tolerance and 46.2% diabetes mellitus. SFRP4 concentrations correlated with insulin (R = 0.153, p = 0.001), HbA1c (R = 0.166, p < 0.0001), fasting triglycerides (R = 0.113, p = 0.011) and higher triglycerides after lipid challenge (postprandial triglycerides R = 0.124, p = 0.005; AUC R = 0.134, p = 0.003). Higher SFRP4 concentrations were associated with type 2 diabetes, metabolic syndrome, and severity of diabetes.

The primary outcome was the composite of cardiovascular death and cardiovascular hospitalization within 48 months follow-up. Comparison of event-free survival between SFRP4 tertiles showed that SFRP4 concentrations were not predictive for cardiovascular outcome.

**Conclusions:**

SFRP4 concentrations are associated with impaired glucose and triglyceride metabolism but do not predict cardiovascular outcome in patients with stable coronary artery disease on treatment.

## Background

Type 2 diabetes (T2DM) is a chronic, progressive disease characterized by insulin resistance and beta-cell dysfunction resulting in the decline of insulin production and secretion. T2DM is contributing to a significant morbidity and mortality, it affects more than 300 million individuals worldwide and the prevalence is still increasing, with the expectation of 439 million adults affected by 2030 [1].

Wnt (**W**ingless and I**nt***-1*) signalling is a conserved pathway involved in embryonic development, the self-renewal of adult tissue and in carcinogenesis [2,3]. Several Wnt pathway components are associated with lipid and glucose metabolism and thereby influence the development of diabetes. Variations in the Wnt co-receptors LRP5 and LRP6 are associated with type 1 diabetes [4] and early coronary disease and the metabolic syndrome [5]. TCF7L2, a transcription factor at the end of the Wnt-signalling cascade is the most prominent hit in genome-wide association studies exploring the genetics of T2DM [6]. TCF7L2 might be a key regulator of proinsulin synthesis, processing and possibly clearance [7] and is involved in the regulation of incretin production [8].

Wnt-signalling is modulated at several levels. A number of secreted proteins, including secreted frizzled-related proteins (SFRPs), bind Wnts, thereby modulating their action [2]. SFRPs share homology with the extracellular domain of frizzled proteins but lack the transmembrane and intracellular components that are necessary for signalling transduction. SFRPs antagonize Wnt-signalling by competitively binding to Wnts or to their receptors in the plasma membrane. In humans, the SFRP family consists of 5 members, termed SFRP1-5. Several SFRPs have been described as adipokines with different roles in adipogenesis [9-11] and SFRP4 as a regulator of insulin exocytosis in murine and human islet cells [12]. Moreover, the authors presented data showing an association of SFRP4 serum levels with insulin resistance and T2DM. Interestingly, in two small cohorts SFRP4 was elevated several years before clinical diagnosis of diabetes, assuming the possibility of SFRP4 as an early diabetes marker [12].

Another SFRP, which has been associated with type 2 diabetes, is SFRP5. However, the role of SFRP5 in type 2 diabetes is still controversial. Three studies describe an association of higher SFRP5 levels with type 2 diabetes [13,14] or insulin resistance in non-diabetic subjects [15], whereas two studies show the opposite effect, lower SFRP5 levels in subjects with prediabetes or type 2 diabetes in comparison to controls [16,17].

To assess the above described association of SFRP4 serum levels with insulin resistance and T2DM we analysed SFRP4 in 504 patients with clinically stable coronary artery disease from the prospective observational Homburg Cream and Sugar study [18]. The strength of the Homburg Cream & Sugar Study is the detailed clinical and metabolic characterisation of the patients, including a sophisticated metabolic test protocol to analyse glucose and triglyceride metabolism.

## Methods

### Study design

Methods of the Homburg Cream and Sugar study (NCT00628524) have been described in detail [18] elsewhere. Briefly, from February 2008 to July 2009, 504 consecutive patients with clinically stable coronary artery disease (CAD) documented by angiography were enrolled in this prospective, observational study. Institutional approval was provided by the ethics committee of the Saarland (number 170/07) and all participants gave written informed consent.

Metabolic syndrome, impaired glucose tolerance and diabetes were defined according to current recommendations [19,20]: The metabolic syndrome was considered when three of the following criteria were positive: elevated waist circumference (men ≥94 cm, women ≥80 cm), elevated fasting TG (≥150 mg/dl), low HDL-cholesterol (men <40 mg/dl, women <50 mg/dl), elevated fasting glucose (≥110 mg/dl or drug treatment for diabetes) or elevated blood pressure (≥140 mmHg systolic or ≥90 mmHg diastolic treatment naïve or on anti-hypertensive treatment). Type 2 diabetes was defined based on validated physician diagnosis and newly diagnosed diabetes according to the guidelines of the American Diabetes Association when fasting glucose was ≥126 mg/dl, HbA1c >6.5% and/or 2-hour glucose was ≥200 mg/l. Laboratory measurements were performed at the core facilities of the Medical University of the Saarland.

Follow-up data were obtained by standardized telephone interviews after 12, 24, and 48 months. The primary outcome was the composite of cardiovascular death and cardiovascular hospitalisation for acute coronary syndrome (for definitions see [21,22]) or hospitalization for unplanned, symptom-induced coronary angiography and revascularization including bypass surgery.

### SFRP4 assay

Fasting SFRP4 concentrations were measured in duplicate by an enzyme-linked immunosorbent assay produced by Phadia GmbH (Freiburg, Germany) in diluted (1:11) fasting serum samples according to the manufacturer’s instructions. A 4–point standard curve (0,0 – 0,2 – 1,0 – 5,0 ng/ml) was created using lyophilized recombinant human SFRP4. Samples that were out of the range of the standard curve were further diluted (1:5) and measured again. Intra-assay and inter-assay variations were 6.8% and 9.1%, respectively.

### Statistical analysis

**Figure 1:**
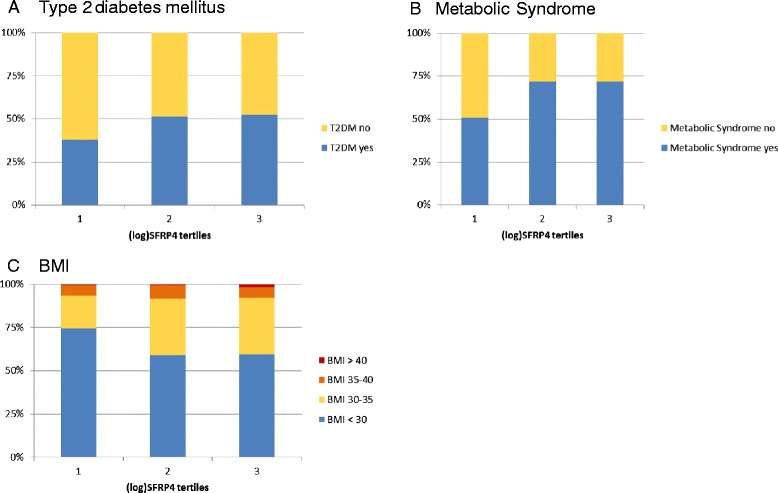
**Incidence of metabolic conditions within (log)SFRP4 tertiles. (A)** Incidence of T2DM within (log)SFRP4 tertiles. **(B)** Incidence of metabolic syndrome within (log)SFRP4 tertiles. **(C)** Incidence of different BMI classes (<30, 30-35, 35-40, >40) within (log)SFRP4 tertiles.

Kolmogorov–Smirnov tests showed that continuous baseline variables were not normally distributed in the cohort. Data are there foreshown as median and interquartile (25%-75%) range (Table 1). SFRP4 serum concentrations were not evenly distributed; therefore, log(SFRP4) was used for all analyses. Patients were stratified by log(SFRP4) tertiles to analyse differences in baseline characteristics using ANOVA for continuous and Fisher’s exact tests for categorical variables (Table 2). Furthermore, log(SFRP4) concentrations were compared with continuous variables by two-sided Pearson correlation and linear regression analyses and between strata of categorical variables by Spearman’s rank correlation (Tables 3 and 4). The number of endpoints between log(SFRP4) tertiles was compared in cross-tables using Chi2 tests (Table 5) and time-to-event analyses were performed with Kaplan-Meier-Curves using log-rank tests (Figure 1) and univariate Cox proportional regression analyses to calculate hazard ratios (HR) and 95% confidence intervals (95%-CI). To this end, the lower log(SFRP4) tertile was set as reference category (HR 1.0) and the upper tertile as the comparator (Table 6).

**Table 1:** **Baseline characteristics of the study cohort**

	**Percentage (number) median (IQR)**
**N**	504
**Age (years)**	68 (59, 74)
**Male**	83.3 (420)
***Clinical characteristics***	
**Smoking (active)**	18.8 (99)
**Alcohol regularly**	23.8 (120)
**Positive family history**	31 (156)
**Hyperlipidemia**	86.3 (435)
**Diabetes mellitus (treated)**	32.7 (165)
**Arterial hypertension**	92.7 (467)
**Systolic blood pressure (mm Hg)**	125 (120, 140)
**Diastolic blood pressure (mm Hg)**	80 (70, 80)
**Body mass index (kg/m** ^**2**^ **)**	28 (26, 31)
**Waist circumference (cm)**	102 (73, 139)
**Waist-to-hip ratio**	1.0 (0.96, 1.05)
***Metabolic characterization***	
**Normal glucose tolerance**	24.4 (123)
**Impaired glucose tolerance**	28.4 (143)
**Diabetes mellitus (treated & newly diagnosed)**	47.2 (238)
**Metabolic syndrome**	64.9 (327)
**Fasting glucose (mg/dl)**	110 (101, 132)
HOMA index (mg/dl*μIU/ml)	2.3 (1.3, 4.1)
HbA1c (%)*	5.8 (5.5, 6.5)
**Total cholesterol (mg/dl)**	167 (143, 199)
**HDL cholesterol (mg/dl)**	43 (35, 53)
**LDL cholesterol (mg/dl)**	100 (80, 129)
**Fasting triglycerides (mg/dl)**	126 (96, 175)
**Postprandial triglycerides, maximum (mg/dl)**	237 (175, 347)
**Postprandial triglycerides, AUC (mg/dl)**	905 (665, 1278)
**C-reactive protein (mg/l)**	2.2 (0.9, 4.9)
**SFRP4 (μg/l)**	11.21 (9.17, 13.86)

Categorical variables are shown as rate (number). Plus-minus values are depicted as median (interquartile range). Active smoking was regular tobacco use at or within 12 months prior to enrolment. Regular alcohol use was consumption of any alcoholic beverage >3 times/week. The HOMA (Homeostasis Model Assessment) index is the fasting glucose concentration (in milligrams per deciliter) multiplied by the fasting insulin concentration (in microunits per milliliter) divided by 405.

*HbA1c was missing in 23 patients.

**Table 2:** **Baseline characteristics, stratified by tertiles of (log)SFRP4**

**(log) SFRP4 Tertiles**				
	**1**	**2**	**3**	
**(log)SFRP4 range**	**<0.99**	**0.99 – 1.11**	**>1.11**	***p-Value***
Group size	33.3 (168)	33.3 (168)	33.3 (168)	
Age (years)	65.2 ± 0.7	65.8 ± 0.8	68.2 ± 0.8	**0.017**
Male	86.3 (145)	85.7 (144)	78.0 (131)	0.068
***Medical history***				
Previous myocardial infarction	35.7 (60)	44.6 (75)	51.8 (87)	**0.014**
PCI	57.1 (96)	66.7 (112)	76.8 (129)	**0.001**
Bypass operation	7.1 (12)	14.3 (24)	16.1 (27)	**0.03**
Previous stroke or TIA	6.0 (10)	12.5 (21)	13.1 (22)	0.065
Peripheral artery disease	6.5 (11)	10.1 (17)	10.7 (18)	0.37
***Clinical characteristics***				
Smoking	21.4 (36)	20.8 (35)	14.3 (24)	0.18
Alcohol regularly	27.4 (46)	25.0 (42)	19.0 (32)	0.18
Positive family history	31.5 (53)	29.2 (49)	32.1 (54)	0.79
Arterial hypertension	88.7 (149)	94.0 (158)	95.2 (160)	0.095
Systolic BP (mmHg)	124.3 ± 1.2	127.0 ± 1.2	128.2 ± 1.3	0.067
Diastolic BP (mmHg)	74.0 ± 0.7	74.9 ± 0.7	74.8 ± 0.8	0.62
BMI (kg/m^2^)	27.9 ± 0.3	29.4 ± 0.3	29.3 ± 0.4	**0.002**
Waist circumference (cm)	100.9 ± 0.8	104.9 ± 0.9	104.9 ± 0.9	**0.001**
Waist-to-hip-ratio	1.00 ± 0.0	1.00 ± 0.0	1.01 ± 0.0	0.36
***Metabolic characterization***				
Normal glucose tolerance	32.1 (54)	20.2 (34)	20.8 (35)	**0.017**
Impaired glucose tolerance	29.8 (50)	28.6 (48)	26.8 (45)	0.84
Diabetes mellitus	38.1 (64)	51.2 (86)	52.4 (88)	**0.014**
Metabolic syndrome	50.6 (85)	72.0 (121)	72.0 (121)	**<0.0001**
Fasting glucose (mg/dl)	118.6 ± 2.5	119.5 ± 2.3	125.0 ± 2.6	0.15
Fasting insulin (μIU/ml)	8.73 ± 0.6	11.41 ± 0.9	12.69 ± 1.1	**0.007**
HOMA index	2.70 ± 0.24	3.64 ± 0.36	4.33 ± 0.55	**0.018**
HbA1c (%)*	6.05 ± 0.1	6.09 ± 0.1	6.40 ± 0.1	**0.011**
Total cholesterol (mg/dl)	175.0 ± 2.8	171.2 ± 3.2	170.9 ± 3.0	0.55
HDL cholesterol (mg/dl)	46.5 ± 1.0	43.8 ± 1.0	43.8 ± 1.1	0.12
LDL cholesterol (mg/dl)	107.7 ± 2.4	105.3 ± 2.8	101.0 ± 2.6	0.19
Fasting triglycerides (mg/dl)	133.9 ± 5.3	164.6 ± 15.0	170.0 ± 9.9	**0.043**
Postprandial triglyerides, maximum (mg/dl)	253.5 ± 9.7	305.4 ± 19.7	309.9 ± 14.2	**0.015**
Postprandial triglycerides, AUC (mg/dl)	943.8 ± 36.2	1135.6 ± 68.5	1160.3 ± 54.4	**0.010**
C-reactive protein (mg/l)	3.65 ± 0.5	4.90 ± 0.5	4.99 ± 0.7	0.16

Categorical variables are shown as rate (number) and continuous values as mean (SEM). TIA denotes transitory ischemic attack. Active smoking was regular tobacco use at or within 12 months prior to enrolment. Regular alcohol use was consumption of any alcoholic beverage >3 times/week. The HOMA (Homeostasis Model Assessment) index is the fasting glucose concentration (in milligrams per deciliter) multiplied by the fasting insulin concentration (in microunits per milliliter) divided by 405.

*HbA1c was missing in 23 patients.

**Table 3:** **Correlation of (log)SFRP4 concentration with baseline characteristics**

**Parameter**	**Pearson R**	**p-Value**
HbA1c	0.17	<0.0001
Fasting insulin	0.15	0.001
Body mass index	0.15	0.001
Fasting triglycerides	0.11	0.011
Postprandial triglycerides	0.12	0.005
Postprandial triglycerides AUC	0.13	0.003
Age	0.09	0.053

Continuous baseline variables were compared with (log)SFRP4 concentrations by two-sided Pearson correlation; no significant association between SFRP4 and other than the listed was seen.

**Table 4:** **Correlation of (log)SFRP4 concentration with baseline characteristics**

**Characteristic**	**No.**	**Log(SRFP4)**	**Spearman’s Rho**	**p-Value**
		**Mean ± SEM**		
Previous myocardial infarction			0.13	0.007
No	282	1.04 ± 0.1		
Yes	222	1.08 ± 0.1		
Previous PCI			0.17	0.001
No	167	1.03 ± 0.1		
Yes	337	1.07 ± 0.1		
Previous cardiac bypass operation			0.13	0.005
No	441	1.05 ± 0.1		
Yes	63	1.10 ± 0.1		
Previous stroke			0.11	0.026
No	451	1.05 ± 0.1		
Yes	53	1.10 ± 0.2		
T2DM			0.11	0.011
No	266	1.04 ± 0.1		
Yes	238	1.07 ± 0.1		
Insulin therapy			0.17	0.001
No	429	1.05 ± 0.1		
Yes	75	1.10 ± 0.2		
Metabolic syndrome			0.16	<0.0001
No	177	1.02 ± 0.1		
Yes	327	1.08 ± 0.1		

Mean (log)SFRP4 concentrations were compared between strata of categorical variables by Spearman’s rank correlation test; no association between SFRP4 and parameters other than the listed.

**Table 5:** **Number of events, stratified by (log)SFRP4 tertiles**

**(log)SFRP4 tertiles**				
	**<0.99**	**0.99 – 1.11**	**>1.11**	**p-Value**
	**N = 168**	**N = 168**	**N = 168**	
**Event number per tertile**				
All primary endpoints	72	69	76	0.74
Acute coronary syndrome	16	15	26	0.11
MACE*	45	40	42	0.82
Stroke/TIA	8	10	19	**0.049**
Cardiovascular death + non-fatal MI	16	20	16	0.71
All-cause death	9	16	18	0.18

Descriptive statistics (Chi2) were used to compare the number of patients with events between (log)SFRP4 tertiles.

*MACE = Major adverse cardiovascular events (Combination of myocardial infarction, unplanned revascularization and cardiovascular death).

**Table 6:** **Number of events, stratified by (log)SFRP4 tertiles**

	**HR**	**95%-CI**	**p-Value**
All primary endpoints	1.05	0.76 - 1.45	0.77
Acute coronary syndrome	1.65	0.89 – 3.08	0.12
MACE*	0.91	0.60 – 1.38	0.64
Stroke/TIA	2.45	1.07 – 5.59	**0.034**
Cardiovascular death + non-fatal MI	0.99	0.50 – 1.99	0.99
All-cause death	2.02	0.91 – 4.50	0.084

Univariate cox proportional hazards regression analyses were used to compare time to events between (log)SFRP4 tertiles. Hazard ratios (HR) and 95% confidence intervals (95%-CI) are provided for the comparison of the third tertile vs. the first tertile (reference category, HR =1.0).

*MACE = Major adverse cardiovascular events (Combination of myocardial infarction, unplanned revascularization and cardiovascular death).

The null hypothesis was rejected and statistical significance was assumed at p-values <0.05. All statistical analyses were performed with SPSS software version 20.0.

## Results

For this study N = 504 patients with angiographically confirmed stable CAD were enrolled. The median age was 68 years and 83% of the patients were male. 32.7% of the patients had a history of T2DM, after oral glucose tolerance test 13.5% (N = 68) were newly diagnosed for T2DM and an impaired glucose tolerance was seen in 29.4%, thus in this cohort only 24.4% of the patients showed a normal glucose tolerance (Table 1). SFRP4 serum levels were in the range of 3.64 – 41.2 μg/l, with a median of 11.21 μg/l. SFRP4 serum concentrations were not evenly distributed (Kolmogorov-Smirnov-Z 4.854, p <0.001); therefore we used (log)SFRP4 for the following analysis.

The patient characteristics in the SFRP4 tertiles are displayed in Table 2 and Figure 1. Patients in the highest SFRP4 tertile were older and more often had a history of myocardial infarction, percutaneous intervention or bypass operation. Waist circumference and BMI were increasing significantly from the lowest to the highest tertile. Patients in the first tertile showed significantly lower levels of fasting insulin, HbA1c, fasting and postprandial triglycerides and in accordance to this the fraction of patients with T2DM or metabolic syndrome in the first tertile was considerably lower in comparison to the other tertiles. In correlation analyses (Table 3) SFRP4 concentrations correlated with HbA1c, fasting insulin, BMI, fasting and postprandial triglycerides. Comparison of SFRP4 concentrations between strata of categorical variables showed that SFRP4 levels were higher in patients with metabolic syndrome, insulin therapy, diabetes and a history of myocardial infarction or PCI (Table 4).

Within 4 years follow-up 287 patients survived event-free and 217 patients experienced a primary cardiovascular endpoint such as acute coronary syndromes, major adverse cardiovascular events and cardiovascular death + non-fatal myocardial infarction. Interestingly, the number of patients experiencing a stroke/transitory ischemic attack (TIA) was significantly higher in the upper SFRP4 tertile, both in descriptive as well as in univariate time-to-event analyses (Tables 5 and 6). However, the association of sFPR4 was not independent of other risk factors, because significance was rapidly lost upon multivariable adjustment (not shown).

## Discussion

The importance and burden of T2DM for our health system is permanently increasing, despite all efforts in primary prevention. Because it is a progressive disease which does not cause specific symptoms for many years diagnosis at an early state is of outmost importance. Usually, the diagnosis starts with measurement of fasting plasma glucose or HbA1c, the advantages and disadvantages of HbA1c measurement are discussed in a WHO report from 2011 [23]. Oral glucose tolerance test is still the gold standard but due to the time consuming procedure not feasible everywhere. With the help of the OGTT we were able to detect 68 (13.5%) previously unknown patients with diabetes in the HCS study, supporting the observation of a high estimated number of unreported cases in secondary prevention cohorts and the general population [24].

Until now unique markers for the different stages of impaired glucose metabolism are missing, except post-prandial plasma glucose. Thus, a recent publication of Mahdi et al. [12] attracted a great deal of attention, cumulating in the title “Mining Genes in Type 2 Diabetic Islets and Finding Gold” of an accompanying editorial [25] and in a computer aided screening for SFRP4 inhibitors [26]. In a set of elegant experiments Mahdi et al. showed that SFRP4 impairs insulin release both *in vitro* in mouse and human islets and *in vivo* in SFRP4-treated mice. The reduced secretion was explained by decreased expression of L-type and P/Q-type Ca2+ channels in the islets’ cells causing a suppression of insulin exocytosis. This corresponds well to previous published data of Taneera et al. [27], describing a significant inverse correlation of SFRP4 expression in human pancreatic islets with insulin secretion (R = −0.28; p = 0.03). This was supported by *in vitro* experiments with isolated human pancreatic islets showing that recombinant SFRP4 inhibits insulin secretion by 30% and cell exocytosis by 50%. Besides the functional characterization of SFRP4 action in islets Mahdi et al. reported a significant correlation of serum SFRP4 concentration with fasting glucose (β = 0.142; p = 0.004), reduced insulin sensitivity index (β = −0.176; p = 0.002) and lower disposition index (insulin secretion adjusted for insulin sensitivity; β = −0.186; p = 0.029) in non-diabetic subjects [12]. Furthermore they described elevated SFRP4 serum levels several years before the clinical diagnosis of T2DM was made, proposing the possibility of SFRP4 as an early risk predictor [12].

In the HCS study we could confirm their observation that T2DM patients are characterized by higher SFRP4 levels. Looking at specific parameters of the glucose metabolism in the HCS study we found for fasting glucose only an insignificant trend towards higher levels in the second and third tertile, whereas we observed a significant positive correlation of SFRP4 serum levels with fasting insulin and HbA1c, a more reliable glucose sensor than fasting glucose. This observation is in part supported by Taneera et al. who described a strong correlation of SFRP4 expression in isolated islet cells with HbA1c levels of the donors [27]. On the other hand at the moment it is not clear to which extend SFRP4 production in islets corresponds to SFRP4 serum levels or vice versa.

We not only observe an association of higher SFRP4 concentrations with T2DM but also with the metabolic syndrome. SFRP4 was associated with higher BMI, waist circumference and triglycerides (fasting as well as postprandial after a standardized lipid challenge), all attributes of the metabolic syndrome. Recently, it has been shown that SFRP4 is an adipokine [11]. The expression of SFRP4 is up-regulated in human visceral white adipose tissue of obese subjects and correlates with increased insulin resistance. There is some evidence that SFRP4 might influence the secretion of adiponectin from adipocytes [11]. SFRP4 is also involved in adipogenesis [9]. Park et al. showed that the expression of SFRP4 is increased during the adipogenic differentiation of human adipose tissue-derived mesenchymal stem cells and that transfection with siSFRP4 reduced the degree of adipocytic differentiation.

A trigger for the increased expression of SFRP4 in diabetes can be methylglyoxal. Methylglyoxal (MG), also called pyruvaldehyde or 2-oxopropanal, is formed by the degradation of the glycolytic intermediates, dihydroxyacetone phosphate, and glyceraldehyde-3-phosphate [28]. MG reacts with free amino groups of lysine and arginine and with thiol groups of cysteine, forming advanced glycation endproducts. MG concentrations are highly increased in diabetes and are associated with the development of diabetic complications, as demonstrated in several studies [29-32]. Recently, Mori et al. [33] could show that MG can increase SFRP4 gene expression 4-fold in ST2 cells, a mouse bone marrow stromal cell-line. This increase was achieved by an epigenetic derepression of the SFRP4 gene.

Studies describing SFRP4 levels in blood are rare; most groups analyzed SFRP4 on the cellular level or within tumor tissues, supporting the function of SFRP4 as tumor suppressor gene [3]. Besides the study of Madhi et al. [12] and the here presented HCS study only three other groups published SFRP4 serum or plasma levels [34-36]. Berndt et al. described for serum a range of 5.5–79.8 ng/ml in five healthy controls [35], Simpson et al. published for serum a mean of 28.5 ± 1.7 ng/ml for 24 unaffected controls and 38.1 ± 2.3 ng/ml for patients with high bone mass causing mutations in LRP5 [36]. Jacob et al. were interested in the role of SFRP4 in ovarian cancer and next to SFRP4 expression levels in tumors and cell-lines they published SFRP4 plasma levels which are about 100-fold higher than in the other studies. This discrepancy underlines the need for standardized assays for the necessary studies to further evaluate the role of SFRP4 in diabetes. Besides, the study of Jacob et al. shows that the interpretation of SFRP4 levels demand a detailed evaluation of the patients: In patients with ovarian cancer the reduction in SFRP4 levels could cover the increase associated with the development of diabetes whereas in diabetic patients the increased SFRP4 levels could interfere with the effort to predict the outcome of a newly diagnosed tumor. Due to the exclusion criteria of the HCS study we can be sure that our results were not compromised by cancer.

Within 4 years follow-up nearly half of the patients experienced a primary cardiovascular endpoint. The association of higher SFRP4 concentrations with stroke/TIA seems to be a hit by chance since significance was rapidly lost upon multivariable adjustment.

### Limitations

During follow-up we obtained no fresh blood samples to diagnose the onset of T2DM or a change in SFRP4 concentration. The number of patients with a new onset of T2DM during follow-up was too small for reliable statistical analysis of this parameter.

## Conclusions

This prospective study shows that higher SFRP4 concentrations are associated with T2DM and the metabolic syndrome in well-treated patients with coronary artery disease. SFRP4 concentrations are a novel marker of impaired glucose and triglyceride metabolism, but do not predict cardiovascular outcome in patients with stable coronary artery disease.

Further research is necessary to elucidate the relevance of SFRP4 levels in healthy people and patients with cardiovascular disease and its prognostic value for diabetes and cancer.

## Abbreviations

AUC: Area under the curve
BMI: Body mass index
BP: Blood pressure
HCS: Homburg cream & sugar study
HOMA: Homeostasis model assessment
MACE: Major adverse cardiovascular events
OGTT: Oral glucose tolerance test
PCI: Percutaneous coronary intervention
SFRP4: Secreted frizzled related protein 4
T2DM: Type 2 diabetes mellitus
TIA: Transitory ischemic attack
Wnt: Wingless and Int-1

## References

[1] Shaw JE, Sicree RA, Zimmet PZ (2010). Global estimates of the prevalence of diabetes for 2010 and 2030. Diabetes Res Clin Pract.

[2] Chien AJ, Conrad WH, Moon RT (2009). A Wnt survival guide: from flies to human disease. J Investig Dermatol.

[3] Surana R, Sikka S, Cai W, Shin EM, Warrier SR, Tan HJ, Arfuso F, Fox SA, Dharmarajan AM, Kumar AP (2014). Secreted frizzled related proteins: implications in cancers. Biochim Biophys Acta.

[4] Twells RC, Mein CA, Payne F, Veijola R, Gilbey M, Bright M, Timms A, Nakagawa Y, Snook H, Nutland S, Rance HE, Carr P, Dudbridge F, Cordell HJ, Cooper J, Tuomilehto-Wolf E, Tuomilehto J, Phillips M, Metzker M, Hess JF, Todd JA (2003). Linkage and association mapping of the LRP5 locus on chromosome 11q13 in type 1 diabetes. Hum Genet.

[5] Mani A, Radhakrishnan J, Wang H, Mani MA, Nelson-Williams C, Carew KS, Mane S, Najmabadi H, Wu D, Lifton RP (2007). LRP6 mutation in a family with early coronary disease and metabolic risk factors. Science.

[6] Grant SF, Thorleifsson G, Reynisdottir I, Benediktsson R, Manolescu A, Sainz J, Helgason A, Stefansson H, Emilsson V, Helgadottir A, Styrkarsdottir U, Magnusson KP, Walters GB, Palsdottir E, Jonsdottir T, Gudmundsdottir T, Gylfason A, Saemundsdottir J, Wilensky RL, Reilly MP, Rader DJ, Bagger Y, Christiansen C, Gudnason V, Sigurdsson G, Thorsteinsdottir U, Gulcher JR, Kong A, Stefansson K (2006). Variant of transcription factor 7-like 2 (TCF7L2) gene confers risk of type 2 diabetes. Nat Genet.

[7] Zhou Y, Park SY, Su J, Bailey K, Ottosson-Laakso E, Shcherbina L, Oskolkov N, Zhang E, Thevenin T, Fadista J, Bennet H, Vikman P, Wierup N, Fex M, Rung J, Wollheim C, Nobrega M, Renström E, Groop L, Hansson O: **TCF7L2 is a master regulator of insulin production and processing**. *Hum Mol Genet* 2014. [Epub ahead of print]. 10.1093/hmg/ddu359 PMC4240194 25015099

[8] Chiang YT, Ip W, Jin T (2012). The role of the Wnt signaling pathway in incretin hormone production and function. Front Physiol.

[9] Park JR, Jung JW, Lee YS, Kang KS (2008). The roles of Wnt antagonists Dkk1 and sFRP4 during adipogenesis of human adipose tissue-derived mesenchymal stem cells. Cell Prolif.

[10] Ouchi N, Higuchi A, Ohashi K, Oshima Y, Gokce N, Shibata R, Akasaki Y, Shimono A, Walsh K (2010). Sfrp5 is an anti-inflammatory adipokine that modulates metabolic dysfunction in obesity. Science.

[11] Ehrlund A, Mejhert N, Lorente-Cebrian S, Astrom G, Dahlman I, Laurencikiene J, Ryden M (2013). Characterization of the Wnt inhibitors secreted frizzled-related proteins (SFRPs) in human adipose tissue. J Clin Endocrinol Metab.

[12] Mahdi T, Hanzelmann S, Salehi A, Muhammed SJ, Reinbothe TM, Tang Y, Axelsson AS, Zhou Y, Jing X, Almgren P, Krus U, Taneera J, Blom AM, Lyssenko V, Esguerra JL, Hansson O, Eliasson L, Derry J, Zhang E, Wollheim CB, Groop L, Renström E, Rosengren AH (2012). Secreted frizzled-related protein 4 reduces insulin secretion and is overexpressed in type 2 diabetes. Cell Metab.

[13] Lu YC, Wang CP, Hsu CC, Chiu CA, Yu TH, Hung WC, Lu LF, Chung FM, Tsai IT, Lin HC, Lee YJ (2013). Circulating secreted frizzled-related protein 5 (Sfrp5) and wingless-type MMTV integration site family member 5a (Wnt5a) levels in patients with type 2 diabetes mellitus. Diabetes Metab Res Rev.

[14] Canivell S, Rebuffat S, E GR, Kostov B, Siso-Almirall A, Novials A, Ceriello A, Gomis R: **Circulating SFRP5 levels are elevated in drug-naive recently diagnosed type 2 diabetic patients as compared with prediabetic subjects and controls**. *Diabetes Metab Res Rev* 2014. doi:10.1002/dmrr.2599. [Epub ahead of print]. 10.1002/dmrr.2599 25139699

[15] Carstensen M, Herder C, Kempf K, Erlund I, Martin S, Koenig W, Sundvall J, Bidel S, Kuha S, Roden M, Tuomilehto J (2013). Sfrp5 correlates with insulin resistance and oxidative stress. Eur J Clin Investig.

[16] Hu Z, Deng H, Qu H (2013). Plasma SFRP5 levels are decreased in Chinese subjects with obesity and type 2 diabetes and negatively correlated with parameters of insulin resistance. Diabetes Res Clin Pract.

[17] Hu W, Li L, Yang M, Luo X, Ran W, Liu D, Xiong Z, Liu H, Yang G (2013). Circulating Sfrp5 is a signature of obesity-related metabolic disorders and is regulated by glucose and liraglutide in humans. J Clin Endocrinol Metab.

[18] Werner C, Filmer A, Fritsch M, Groenewold S, Graber S, Bohm M, Laufs U: **Risk prediction with triglycerides in patients with stable coronary disease on statin treatment.** *Clin Res Cardiol* 2014. [Epub ahead of print]. 10.1007/s00392-014-0740-0 25012240

[19] Alberti KG, Eckel RH, Grundy SM, Zimmet PZ, Cleeman JI, Donato KA, Fruchart JC, James WP, Loria CM, Smith SC (2009). Harmonizing the metabolic syndrome: a joint interim statement of the International Diabetes Federation Task Force on Epidemiology and Prevention; National Heart, Lung, and Blood Institute; American Heart Association; World Heart Federation; International Atherosclerosis Society; and International Association for the Study of Obesity. Circulation.

[20] American Diabetes Association (2011). Executive summary: standards of medical care in diabetes–2011. Diabetes Care.

[21] Thygesen K, Alpert JS, Jaffe AS, Simoons ML, Chaitman BR, White HD, Writing Group on behalf of the Joint ESCAAHAWHFTFftUDoMI (2012). Third universal definition of myocardial infarction. J Am Coll Cardiol.

[22] Anderson JL, Adams CD, Antman EM, Bridges CR, Califf RM, Casey DE, Chavey WE, Fesmire FM, Hochman JS, Levin TN, Lincoff AM, Peterson ED, Theroux P, Wenger NK, Wright RS, Jneid H, Ettinger SM, Ganiats TG, Lincoff AM, Philippides GJ, Zidar JP (2013). 2012 ACCF/AHA focused update incorporated into the ACCF/AHA 2007 guidelines for the management of patients with unstable angina/non-ST-elevation myocardial infarction: a report of the American College of Cardiology Foundation/American Heart Association Task Force on Practice Guidelines. Circulation.

[23] **Abbreviated report of a WHO consultation. Use of glycated hemoglobin (HbA1c) in the diagnosis of diabetes mellitus.** [http://www.who.int/diabetes/publications/diagnosis_diabetes2011/en/] 26158184

[24] DECODE Study Group (2003). Age- and sex-specific prevalences of diabetes and impaired glucose regulation in 13 European cohorts. Diabetes Care.

[25] Eizirik DL, Cnop M (2012). Mining genes in type 2 diabetic islets and finding gold. Cell Metab.

[26] Bukhari SA, Shamshari WA, Ur-Rahman M, Zia-Ul-Haq M, Jaafar HZ (2014). Computer aided screening of Secreted Frizzled-Related Protein 4 (SFRP4): a potential control for diabetes mellitus. Molecules.

[27] Taneera J, Lang S, Sharma A, Fadista J, Zhou Y, Ahlqvist E, Jonsson A, Lyssenko V, Vikman P, Hansson O, Parikh H, Korsgren O, Soni A, Krus U, Zhang E, Jing XJ, Esguerra JL, Wollheim CB, Salehi A, Rosengren A, Renström E, Groop L (2012). A systems genetics approach identifies genes and pathways for type 2 diabetes in human islets. Cell Metab.

[28] Ohmori S, Mori M, Shiraha K, Kawase M (1989). Biosynthesis and degradation of methylglyoxal in animals. Prog Clin Biol Res.

[29] Lu J, Randell E, Han Y, Adeli K, Krahn J, Meng QH (2011). Increased plasma methylglyoxal level, inflammation, and vascular endothelial dysfunction in diabetic nephropathy. Clin Biochem.

[30] Mukohda M, Okada M, Hara Y, Yamawaki H (2012). Exploring mechanisms of diabetes-related macrovascular complications: role of methylglyoxal, a metabolite of glucose on regulation of vascular contractility. J Pharmacol Sci.

[31] Lu J, Ji J, Meng H, Wang D, Jiang B, Liu L, Randell E, Adeli K, Meng QH (2013). The protective effect and underlying mechanism of metformin on neointima formation in fructose-induced insulin resistant rats. Cardiovasc Diabetol.

[32] Su Y, Qadri SM, Wu L, Liu L (2013). Methylglyoxal modulates endothelial nitric oxide synthase-associated functions in EA.hy926 endothelial cells. Cardiovasc Diabetol.

[33] Mori K, Kitazawa R, Kondo T, Mori M, Hamada Y, Nishida M, Minami Y, Haraguchi R, Takahashi Y, Kitazawa S (2014). Diabetic osteopenia by decreased beta-catenin signaling is partly induced by epigenetic derepression of sFRP-4 gene. PLoS ONE.

[34] Jacob F, Ukegjini K, Nixdorf S, Ford CE, Olivier J, Caduff R, Scurry JP, Guertler R, Hornung D, Mueller R, Fink DA, Hacker NF, Heinzelmann-Schwarz VA (2012). Loss of secreted frizzled-related protein 4 correlates with an aggressive phenotype and predicts poor outcome in ovarian cancer patients. PLoS ONE.

[35] Berndt T, Craig TA, Bowe AE, Vassiliadis J, Reczek D, Finnegan R, Jan De Beur SM, Schiavi SC, Kumar R (2003). Secreted frizzled-related protein 4 is a potent tumor-derived phosphaturic agent. J Clin Investig.

[36] Simpson CA, Foer D, Lee GS, Bihuniak J, Sun B, Sullivan R, Belsky J, Insogna KL (2014). Serum levels of sclerostin, Dickkopf-1, and secreted frizzled-related protein-4 are not changed in individuals with high bone mass causing mutations in LRP5. Osteoporos Int.

